# Commentary: Multiscale Analysis of Independent Alzheimer's Cohorts Finds Disruption of Molecular, Genetic, and Clinical Networks by Human Herpesvirus

**DOI:** 10.3389/fnmol.2018.00340

**Published:** 2018-09-20

**Authors:** Xiao-Wen Sun, Chang-Mei Liu, Zhao-Qian Teng

**Affiliations:** ^1^State Key Laboratory of Stem Cell and Reproductive Biology, Institute of Zoology, Chinese Academy of Sciences, Beijing, China; ^2^University of Chinese Academy of Sciences, Beijing, China; ^3^Institute of Stem Cell and Regeneration, Chinese Academy of Sciences, Beijing, China

**Keywords:** alzheier disease, herpes virus, miR-155 inhibition, HHV 6, HHV 7

Alzheimer's disease (AD), the most common type of dementia among the elderly, is caused by progressive neural death that results in impaired memory, thinking skills and, eventually, the ability to carry out simple tasks. Unfortunately, approximately 5% of people over the age of 65 suffer from AD, and the prevalence of AD increases with aging (Udeochu et al., 2018). Pathological deposition and accumulation of β-amyloid (Aβ) into senile plaques and hyperphosphorylated Tau into neurofibrillary tangles are widely acknowledged hallmarks of AD (Frere and Slutsky, 2018). The amyloid cascade hypothesis posits that Aβ is the key trigger of AD pathology (Hardy and Selkoe, 2002; Musiek and Holtzman, 2015; Selkoe and Hardy, 2016). However, following repeated failures of Aβ-targeted medicine therapeutics, it has been argued that Aβ may does not play a prominent role in the symptomatic stages of this disease, or the progression of AD cannot been rescued after the emergence of symptom. Understanding the earliest causal elements of AD is difficult for its borderless and protracted preclinical process, and lack of available staged brain tissue samples (Zhang et al., 2013). Therefore the initiating events and earliest drivers that eventually lead to clinical AD symptoms still remain controversial (Poo et al., 2016).

Investigators have proposed that the onset and progression of AD is contributed by pathogenic microbes although definitive evidence has not been presented (Sjogren et al., 1952; Middleton et al., 1980; Itzhaki, 2014). A recent report published in the June 21 issue of the journal Neuron by Dr. Ben Readhead and colleagues at Icahn School of Medicine at Mount Sinai provides novel evidence that viral species, particularly, particularly human herpesviruses HHV-6A and HHV-7, may have been potential earliest drivers which regulate molecular, clinical, and neuropathological networks of AD. To examine whether viral activity constitutes a general feature of AD, they started to map and compare biological networks underlying the preclinical AD (brains meeting neuropathological criteria for AD from individuals who were cognitively intact at the time of death) using multiple independent datasets collected from human subjects. They found that C2H2 zinc finger transcription factor (C2H2-TF) binding motifs and G-quadruplex (G4) sequences are strongly enriched among the promoters of genes that present only in the preclinical AD network (“Gained in preclinical AD”) and those present only in the network (“Lost in preclinical AD”), suggesting a potential role for virus-mediated network activities in AD. To directly examine viral sequences, they examined four, large multi-omic datasets and observed the presence of many viral species in the aging brain and linked multiple viral species with regulation of AD genetic risk networks, AD gene expression changes, and association with clinical dementia rating and neuropathology burden. By comparing datasets among different independent cohorts or between AD and other neuropathological controls, they found that viral genes in HHV-6A and HHV-7 appear at least partly specific to AD, although HHV-6A may also be relevant to other diseases such as progressive supranuclear palsy (PSP). They then extended their analysis of the association between viral gene RNA abundance and AD-relevant clinical and neuropathological traits, and found miR-155 inhibition by HHV-6A, as described in HHV-6A infected T cells (Caselli et al., 2017). Molecular and functional enrichments of the miR-155-KO differentially expressed genes suggested that miR-155 might play a key role in host response to AD-relevant viral perturbation, and act as a potential mediator of neuronal loss.

Based on unbiased approaches and large-scale data sets from several brain banks and cohort studies, this is the first study to provide strong evidence supporting the controversial hypothesis that viruses play an essential role in regulatory genetic networks that are believed to lead to AD. Identifying links to viruses may help scientists interested in developing potential new treatment strategies.

Several important questions are raised from this phenomenal study. First, does herpes virus cause the onset or progression of AD? Eimer and colleagues recently reported that Aβ traps herpes viruses in insoluble amyloid, and active herpes infections in brain accelerate amyloid deposition, indicating that herpes infection may promote AD pathology directly via amyloid-mediated pathological pathways (Eimer et al., 2018). HHV-6 and -7 are no longer considered benign but are now recognized as significant causes of viral encephalitis, particularly in immunocompromised individuals, and have also been shown to be associated with demyelinating brain diseases and epilepsy (Sellner and Trinka, 2012; Campbell et al., 2017). As this study has identified a clear link between herpes viral DNA sequences and activation of molecular, genetic and clinical aspects of AD, future studies are necessary to explore the nature of this link.

Second, whether herpesviruses regulate, or are regulated by AD-associated genes? Will anti-herpes drugs be effective against early onset of AD? This study established a strong connection between multiple viruses, especially HHV-6A, and AD risk genes, including PSEN1, BACE1, and APBB2 which are implicated in regulation of Aβ production. Besides, several recent studies show that Aβ is an antimicrobial protein of the body's innate immune system, capable of providing immediate, effective protection from infection with pathogens like herpes viruses in both cultured human brain cells and animal models of AD (Kumar et al., 2016; Eimer et al., 2018). Virus-host protein and RNA networks revealed by this study suggest many potentially fruitful avenues for future investigations of mechanism and treatment for AD.

Third, is miR-155 a regulator of anti- or pro-viral activity in early AD? Could miR-155 gain-of-function help with Aβ clearance and neuroprotection in AD? MiR-155 has been reported as an important regulator of T cell and microglia in response to neurodegeneration (Song and Lee, 2015; Krasemann et al., 2017). The miR-155 immune network offers a targeted area for developing effective drugs for treating AD.

Lastly, there remain some open issues that future studies will need to address about the Readhead et al. findings. For example, HHV-6A/B and HHV-7 are considered lymphotropic rather than neurotrophic (Berneman et al., 1992; Mori, 2009). Although neither terms are entirely accurate, this characterization does serve to illustrate these viruses principally target T cells and macrophage. In AD there is significant infiltration into brain by macrophages and T and B cells (Lindsay and Christian, 2015). Thus, it is possible that the viral signal seen by Readhead et al originated in the periphery. This will need to be addressed in future studies.

## Author contributions

X-WS drafted an initial version of this commentary. C-ML and Z-QT revised and finalized the text. All authors approved it for publication.

### Conflict of interest statement

The authors declare that the research was conducted in the absence of any commercial or financial relationships that could be construed as a potential conflict of interest.
